# Parthenolide Inhibits STAT3 Signaling by Covalently Targeting Janus Kinases

**DOI:** 10.3390/molecules23061478

**Published:** 2018-06-19

**Authors:** Man Liu, Chengqian Xiao, Mingwei Sun, Minjia Tan, Lihong Hu, Qiang Yu

**Affiliations:** 1 Shanghai Institute of Materia Medica, Chinese Academy of Sciences, Shanghai 201203, China; liuman@simm.ac.cn; 2 University of Chinese Academy of Sciences, Beijing 100049, China; cmusmw@163.com (M.S.); mjtan@simm.ac.cn (M.T.); 3 Jiangsu Key Laboratory for Functional Substance of Chinese Medicine, Jiangsu Collaborative Innovation Center of Chinese Medicinal Resources Industrialization, Stake Key Laboratory Cultivation Base for TCM Quality and Efficacy, School of Pharmacy, Nanjing University of Chinese Medicine, Nanjing 210023, China; xchq890503@163.com (C.X.); lhhu@njucm.edu.cn (L.H.); 4 State Key Laboratory of Drug Research, Shanghai Institute of Materia Medica, Chinese Academy of Sciences, Shanghai 201203, China; 5 The Chemical Proteomics Center, Shanghai Institute of Materia Medica, Chinese Academy of Sciences, Shanghai 201203, China

**Keywords:** parthenolide, covalent inhibitor, cell death, JAK/STAT3 signaling

## Abstract

Aberrant activations of the STAT3 (signal transducer and activator of transcription 3) signaling pathway are associated with cancer and inflammatory diseases. Three of the four Janus kinases, JAK1, JAK2, and Tyk2, are the major upstream kinases of STAT3 in responses to cytokine stimulations. Among them, JAK2 is the key kinase in the IL-6-induced STAT3 phosphorylation. Here we report the mechanisms of a natural compound parthenolide from the medicinal herb Feverfew in regulating the JAK/STAT3 signaling. We found that parthenolide was a potent inhibitor of JAKs. It covalently modified the Cys178, Cys243, Cys335, and Cys480 of JAK2 and suppressed its kinase activity. It also interacted with other JAKs in a similar fashion. The binding of parthenolide to JAKs was selective. It preferentially bound to the JAKs, but not to the abundant proteins, such as tubulin and actin. Parthenolide also induced reactive oxygen species (ROS), but the increased ROS did not seem to contribute to the inhibition of JAK/STAT3 signaling. Furthermore, parthenolide inhibited the IL-6-induced cancer cell migration and preferentially inhibited the growth of cancer cells that had constitutively activated STAT3. Our study suggests a novel strategy to inactivate JAKs and provides a promising anti-inflammation and anticancer drug candidate.

## 1. Introduction

The JAK/STAT (Janus kinase/signal transducer and activator of transcription) signaling pathways are major pathways for cytokine signaling and regulate important cellular events, such as hematopoiesis and immune development [1,2]. Aberrant STAT3 signaling is frequently linked to cancer cell proliferation, survival, metastasis, tumor immunosuppression, and angiogenesis [3,4,5,6,7]. JAK2 is the major kinase of STAT3 [8] and has been reported to be constitutively active in many cancers and other proliferative diseases [9]. The activating mutation V617F in JAK2 is considered as a driving factor for myeloproliferative disorders [10,11]. Therefore, JAK2 has become an important target for the development of new drugs to treat these diseases [12,13].

Thus far, most of the JAK2 inhibitors developed are ATP-competitive kinase inhibitors, such as CYT387, BMS911543, TG101348, and Ruxolitinib [14,15,16]. Ruxolitinib was the first FDA-approved JAK inhibitor to treat myelofibrosis [17,18,19]. However, it has not achieved significant reductions in disease burden in most patients with myelofibrosis [20]. This failure is caused by the heterodimerization between activated JAK2 and other JAKs, resulting in the reactivation of this pathway. Moreover, CYT387, BMS911543, or TG101348 were cross-persistent to ruxolitinib [21]. These findings suggest that more JAK2 inhibitors with different mechanisms are needed to improve efficacy. In this regard, covalent inhibitors have their unique advantages over ATP-competitive kinase inhibitors, because they can irreversibly interact with their targets and prevent the reactivation of JAK-STAT signaling described above. Furthermore, covalent inhibitors can dissociate drug pharmacodynamics from pharmacokinetics, resulting in desired drug efficacy with short systemic exposure to decrease drug interactions with off-targets. It can also achieve prolonged effects, resulting in less-frequent drug dosing [22]. 

Parthenolide (PN) is an abundant sesquiterpene lactone found in the medicinal herb Feverfew (*Tanacetum parthenium*). It has been used to treat arthritis, fever, headache for centuries in Europe. PN has been reported to exhibit inhibitory activity on the IL-6-induced STAT3 activation, which contributes to its anti-inflammation and anti-cancer properties [23]. However, the mechanisms of PN as an inhibitor of JAK-STAT3 signaling are still unknown. In the present report, we investigated the molecular mechanisms of PN in regulating JAK-STAT3 signaling. Our study provides a covalent strategy to develop a JAK inhibitor and suggests PN as a promising anti-inflammation and anticancer drug candidate. 

## 2. Result

### 2.1. Parthenolide Inhibited the IL-6-Induced STAT3 Phosphorylation by Inhibiting JAK2 Kinase Activity

**Figure 1:**
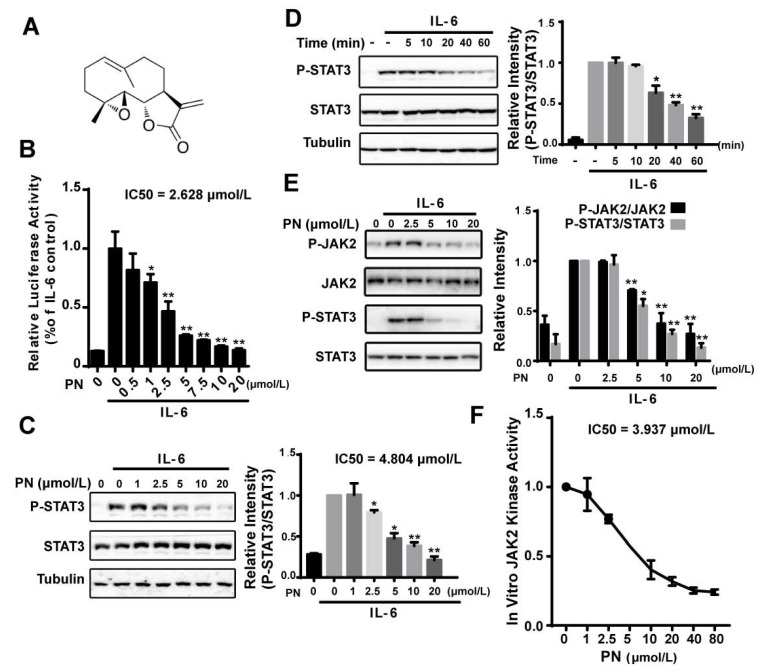
Parthenolide inhibited the IL-6-induced STAT3 phosphorylation by inhibiting JAK2 kinase activity. (**A**) Structure of parthenolide. (**B**) Effects of PN on the IL-6-induced luciferase activity. HepG2/STAT3 cells were treated with PN for 1 h before stimulation by 10 ng/mL IL-6 for 4 h. Cells were harvested to determine the luciferase activity. (**C**,**D**) PN inhibited IL-6-induced STAT3 phosphorylation. MDA-MB-231 cells were treated with PN at various concentrations for 1 h or with 5 μmol/L PN for various durations before stimulation by IL-6 (10 ng/mL) for 10 min. Cell lysates were processed for Western blot analysis. (**E**) Effects of PN on IL-6-induced JAK2 phosphorylation. MEF cells were treated with PN for 1 h before stimulation by IL-6 (10 ng/mL) for 10 min. Cell lysates were processed for Western blot analysis. (**F**) JAK2 in vitro kinase assay. The experiment was conducted as described in the Materials and Methods. (*n* = 3, * *p* < 0.05, ** *p* < 0.01 compared with IL-6-induced cells, one-way ANOVA).

We identified parthenolide (PN) (Figure 1A) as a STAT3 pathway inhibitor from screening a natural compound library using an IL-6–induced STAT3-responsive luciferase reporter assay. PN inhibited the IL-6–induced luciferase activity in a dose-dependent manner (IC50 = 2.628 μmol/L) (Figure 1B). We then examined the effects of PN on the STAT3 Tyr705 phosphorylation, which indicated the activation of STAT3. IL-6–induced STAT3 tyrosine phosphorylation was effectively inhibited by PN with an IC50 of 4.804 μmol/L, and the inhibition occurred 20 min after PN treatment (Figure 1C,D). 

JAK2 has been known to be the major kinase to activate STAT3 in the IL-6 signaling pathway [8]. We, therefore, analyzed the effects of PN on the JAK2 phosphorylation (Y1007/1008) using a murine embryonic fibroblast (MEF) cell line lacking endogenous STAT3 expression, which was used to study JAKs in modulating STAT3 activity [24]. We transiently transfected the MEF cells with a STAT3 expressing plasmid for 24 h. After that, cells were incubated with PN. We found that PN dose-dependently inhibited JAK2 and STAT3 phosphorylation induced by IL-6 (Figure 1E). 

The effects of PN on JAK2 activity in an in vitro kinase assay was then examined using overexpressed and immunoprecipitated JAK2 kinase from HEK293 cells. We found that PN directly inhibited JAK2 activity with an IC50 of 3.937 μmol/L (Figure 1F), demonstrating that PN inhibited the IL-6-induced STAT3 phosphorylation by directly inhibiting JAK2 kinase activity. 

### 2.2. Parthenolide Was a Covalent Pan-JAK Inhibitor

**Figure 2:**
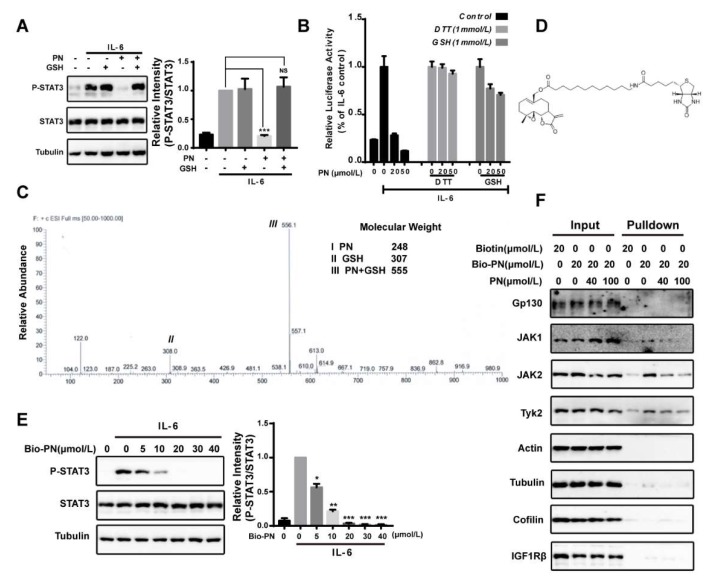
Parthenolide was a pan-JAK covalent inhibitor. (**A**) GSH blocking assay. MDA-MB-231 cells were treated with GSH (1 mmol/L), PN (20 μmol/L) or their incubation products for 1 h and then stimulated with IL-6 (10 ng/mL) for 10 min. Cell lysates were processed for Western blot analysis. (**B**) GSH and DTT blocking assay. HepG2/STAT3 cells were treated with GSH, DTT, PN or their incubation products for 1 h before stimulation by 10 ng/mL IL-6 for 4 h. Cells were harvested for luciferase assay. (**C**) LC-MS analysis of the incubation product of PN and GSH. 0.5 mmol/L PN was incubated with 2 mmol/L of GSH for 1 h at 37 °C. The incubation products were analyzed by LC-MS. Molecular weights of the molecules are indicated. (**D**) The structure of biotinylated parthenolide (Bio-PN). (**E**) Effects of Bio-PN on IL-6-induced STAT3 phosphorylation. MDA-MB-231 cells were treated with Bio-PN for 1 h before stimulation by IL-6 (10 ng/mL) for 10 min. Cell lysates were processed for Western blot analysis. (**F**) Pull-down assay. MDA-MB-231 cells were treated with PN or DMSO for 1 h and then were incubated with Bio-PN or DMSO for 1 h. Streptavidin resin was used for the enrichment of targeted proteins. The precipitates were processed for Western blot analysis. (*n* = 3, NS = No statistical difference, * *p* < 0.05, ** *p* < 0.01, *** *p* < 0.01 compared with IL-6-induced cells, one-way ANOVA).

PN contains an α,β-unsaturated carbonyl group that can potentially react with protein thiols through Michael addition [25,26,27]. To investigate the possibility that PN might interact with JAK2 through covalent modifications of cysteine thiols, we pre-incubated PN with thiol-containing reagents dithiothreitol (DTT) or glutathione (GSH) to block the effects of PN on STAT3. The results demonstrated that the inhibitory effect of PN on STAT3 phosphorylation was abrogated by the pre-incubation treatment (Figure 2A,B). We further analyzed the products of the incubation of PN with GSH by LC-MS and found a major product at *m*/*z* 556 [PN + GSH + H], indicating that one molecule of GSH reacted with one molecule of PN (Figure 2C). 

To identify proteins covalently targeted by PN, we synthesized a biotinylated PN (Bio-PN) to perform a protein pull-down assay (Figure 2D). The Bio-PN retained the biological activities of PN and effectively blocked the IL-6-induced STAT3 phosphorylation at 20 μmol/L (Figure 2E). After incubation with the Bio-PN, the MDA-MB-231 cell lysates were precipitated with streptavidin resin and analyzed by Western blotting. As shown in Figure 2F, JAK1, JAK2 and Tyk2 were pulled down by Bio-PN and could be out-competed by excessive unlabeled PN. Other proteins such as Gp130, the cytokine receptor upstream of JAK2, and tyrosine kinase IGF1Rβ, and several abundant proteins, such as actin, β-tubulin, and cofilin-1, were not detected in the precipitates, suggesting that PN was relatively selective for interacting with JAKs and its effects on JAKs were not the results of promiscuous interactions with proteins.

### 2.3. Parthenolide Covalently Modified Cys178, Cys243, Cys335, and Cys480 of Mouse JAK2

**Figure 3:**
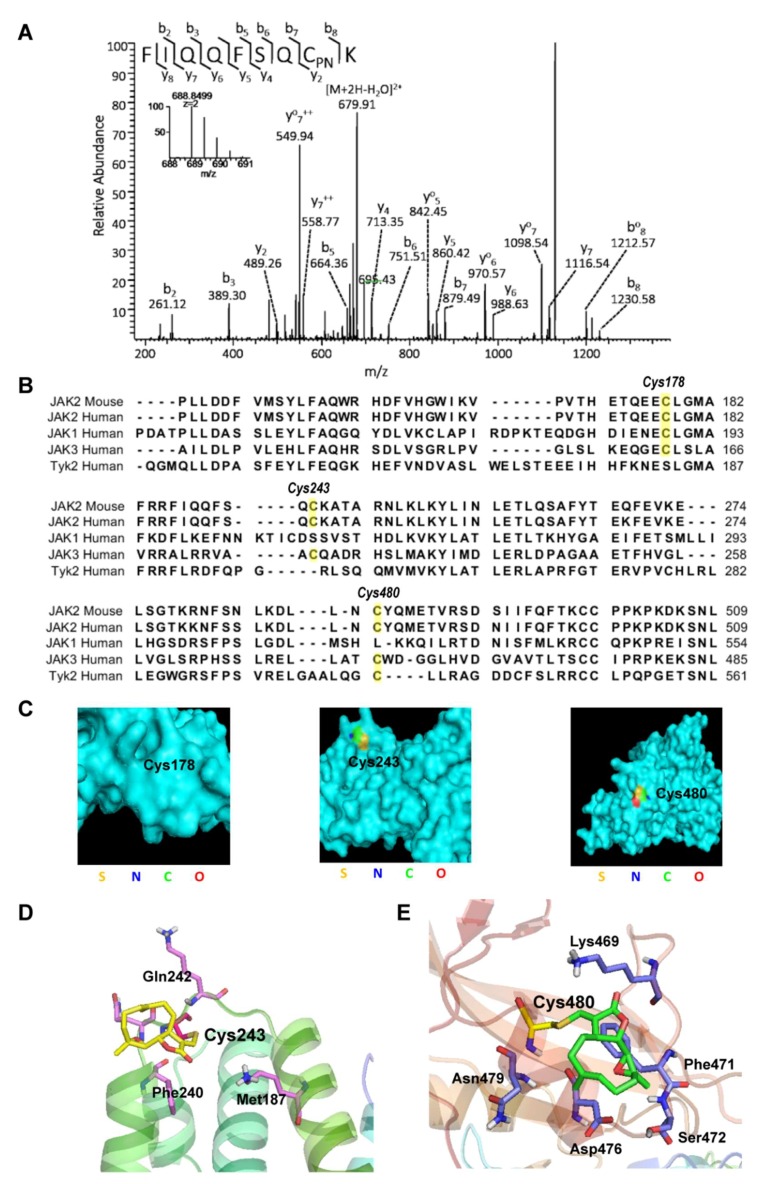
LC-MS/MS and virtual docking analysis of parthenolide-treated JAK2 protein. (**A**) Cys243 of JAK2 was covalently modified by PN. JAK2-overexpressed HEK 293 cells were incubated with 20 μmol/L PN for 1 h. After that, JAK2 protein was immunoprecipitated and subjected to LC-MS/MS analysis. (**B**) Protein sequence alignment of JAK family members. This work was done by the CLC Main Workbench 6.9.1 software package. (**C**) The positions of Cys178, Cys243, and Cys480 on JAK2 protein. The crystal structure of JAK2 (4Z32) was from RCSB and generated by Pymol. Cysteines were colored. (**D**) The binding mode of PN to Cys243. The binding mode shows that the side chain of PN extends to a hydrophobic pocket made up of Met187 and Phe240. The docking result is shown in a cartoon representation (rainbow). PN is shown as yellow sticks and the amino acids that interact with PN are shown as purple sticks. The docking study was performed using Schrödinger program suite as described in Material and Methods. (**E**) The binding mode of PN to Cys480. The docking result is shown in a cartoon representation (light red). PN is shown as green sticks and the amino acids that interact with PN are shown as blue sticks. The binding mode showed that backbone of this compound was close to the hydrophobic residue of Phe471. The docking study was performed using Schrödinger program suite as described in the Materials and Methods.

To determine the amino acid residues modified by PN in JAK2, we transfected the HEK293 cells with a mouse JAK2 cDNA containing plasmid. After incubation with PN, the overexpressed mouse JAK2 protein was immunoprecipitated and subjected to LC-MS/MS analysis. The identification of protein was performed by searching UniProt database using MASCOT algorithm (http://www.matrixscience.com/). The JAK2 protein was identified with 85% sequence coverage (Figure S1). We analyzed all the tryptic peptides from JAK2 and identified four peptides (VPVTHETQEECLGMAVLDMMR, FIQQFSQCK, QANQECSNESR, and DLLNCYQMETVR) with a mass shift of 248.1412 Da that matched the molecular weight of a PN molecule. The identified peptides suggested that PN covalently modified Cys178, Cys243, Cys335, and Cys480 of JAK2 (Figure 3A and Figures S2–S4).

We compared the protein sequence of the mouse JAK2 with the four human JAK family members and found that the Cys178, Cys243, and Cys480 were conserved among the JAK family members, consistent with what we observed from the pull-down assay. PN pulled down JAK1, Tyk2, and JAK2 effectively (Figure 3B). JAK3 was not analyzed in our experiments.

### 2.4. Virtual Docking Demonstrated the Preference of Parthenolide to Cys243 and Cys480 of JAK2

To understand how PN may interact with JAK2, we first examined the locations of the three cysteines on the crystal structure of human JAK2 [28]. As shown in Figure 3C, Cys243 and Cys480 were on the surface of the protein, suggesting the accessibility of Cys243 and Cys480 by PN. To further explore the binding mode of PN, we conducted a virtual molecular docking model using the crystal structure of the FERM and SH2 domain of the human JAK2. We found that Cys243 and Cys480 were successfully docked, while Cys178 was not. The binding mode of PN to Cys243 demonstrated that the side chain of PN extended to a hydrophobic pocket made up of Met187 and Phe240. The binding mode of PN to Cys480 demonstrated that the backbone of this compound was close to the hydrophobic residue of Phe471 (Figure 3D,E). Thus, the virtual docking study explained the preference of PN to Cys243 and Cys480 of JAK2.

### 2.5. The Effects of Parthenolide on JAKs Were Selective

**Figure 4:**
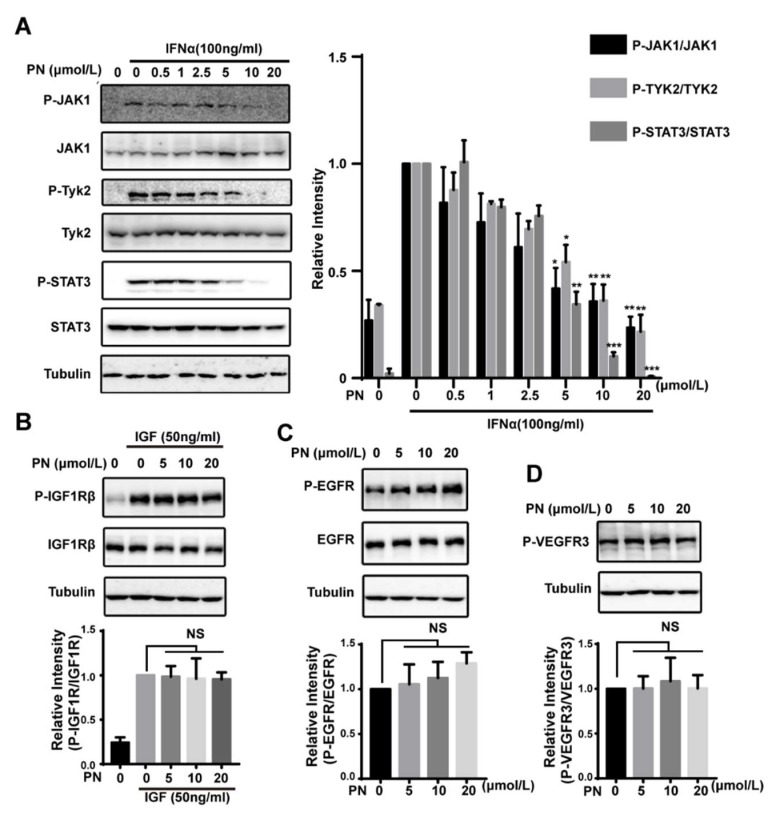
The effects of parthenolide on JAKs were selective. (**A**) Effects of PN on JAK1 and Tyk2 phosphorylation. MDA-MB-231 cells were treated with PN for 1 h before stimulation by IFNα for 10 min. Cell lysates were processed for Western blot analysis. (**B**) Effects of PN on the IGF1-induced IGF1Rβ phosphorylation. MDA-MB-231 cells were incubated with PN for 1 h and stimulated with IGF1 for 10 min. Cell lysates were processed for Western blot analysis. (**C**,**D**) Effects of PN on EGFR and VEGFR3 phosphorylation. MDA-MB-231 cells were incubated with PN for 1 h. Cell lysates were subjected to Western blot analysis. (*n* = 3, NS = no statistical difference, * *p* < 0.05, ** *p* < 0.01, *** *p* < 0.001, One-way ANOVA).

The above data suggested that PN was a pan-JAK inhibitor. To confirm the inhibitory effects of PN on JAK1 and Tyk2 activities, we explored the effects of PN on JAK1/Tyk2 phosphorylation. The IFNα-induced JAK1 and Tyk2 phosphorylation was blocked by PN, as was the phosphorylation of STAT3 (Figure 4A).

To further examine the selectivity of PN, we analyzed the effects of PN on other tyrosine kinases including EGFR, VEGFR3, and IGF-IR and found that PN did not inhibit the phosphorylation of the receptor tyrosine kinases (Figure 4B–D). Using an in vitro kinase assay, we also explored the effects of PN on other kinase activities. The result demonstrated that PN had little inhibitory effects on EGFR, PI3K, c-Src, or MAPK1 at 50 μmol/L (Table 1). These data together confirmed the selectivity of PN on JAKs.

These in vitro kinase assays were performed as described in the Material and Methods.

### 2.6. Reactive Oxygen Species Produced by Parthenolide Did Not Participate in the Inhibition of JAK/STAT3 Signaling

**Figure 5:**
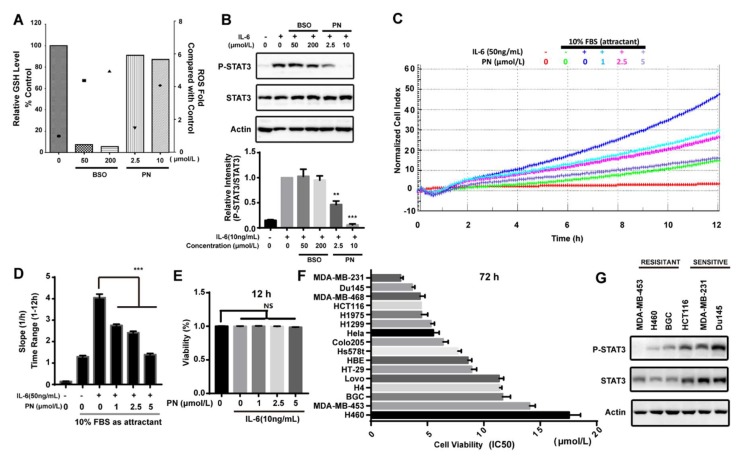
ROS did not participate in the inhibition of STAT3 phosphorylation and parthenolide inhibited the migration and growth of cancer cells. (**A**) The effects of BSO and PN on the cellular GSH and ROS level. MDA-MB-231 cells were treated with BSO for 24 h or with PN for 30 min. Cellular GSH level (histogram) and ROS level (scattergram) were determined as described in Material and Methods. (**B**) The effects of BSO and PN on IL-6-induced STAT3 phosphorylation. MDA-MB-231 cells were treated with BSO for 24 h or with PN for 30 min before stimulation by IL-6 for 10 min. Cell lysates were processed for Western blot analysis. (**C**) Parthenolide inhibited IL-6-induced cancer cell migration. MDA-MB-231 cells were seeded in serum-free medium in the upper chamber. 10% FBS was added in the lower chamber. Cells were then treated with PN. The migration was monitored by real-time cell analysis system for 12 h. (**D**) The migration rate was quantified by RTCA software 2.0. (**E**) The effect of PN on cell viability. Cells were treated with PN for 12 h. The effect on cell viability was determined by alamar blue assay. (**F**) IC50 of PN on growth of different human cancer cell lines. Cells were incubated with PN for 72 h. The drug effect on cell growth was determined by MTT assay. IC50 (means ± SD, *n* = 3). (**G**) The expression and phosphorylation of STAT3 in the sensitive and resistant cancer cells. Cells were lysed and analyzed by Western blot. (*n* = 3, NS = no statistical difference, ** *p* < 0.001, *** *p* < 0.001, One-way ANOVA).

Reactive oxygen species (ROS) was reported to inhibit STAT3 activation [29]. PN has the ability to induce ROS. We assessed the effects of PN-induced ROS on STAT3 phosphorylation induced by IL-6 in MDA-MB-231 cells. BSO (l-buthionine-sulfoximine) is an inhibitor that can inhibit GSH synthesis and accumulate ROS. As shown in Figure 5A, BSO caused the depletion of GSH, and the ROS level generated by BSO at 50 μmol/L was higher than that generated by PN at 10 μmol/L. However, BSO did not affect the STAT3 phosphorylation even at 200 μmol/L while PN completely inhibited STAT3 phosphorylation at 10 μmol/L (Figure 5B). These data suggested that ROS production did not participate in the inhibition of STAT3 activation and that the inhibition on STAT3 phosphorylation was mainly caused by the direct interaction between PN and JAKs.

### 2.7. Parthenolide Selectively Inhibited the IL-6-Induced MDA-MB-231 Migration

IL-6 in a tumor microenvironment is reported to promote cancer cell migration [30,31,32]. Since PN is a JAK2 covalent inhibitor and can inhibit the IL-6-induced STAT3 activation, we next examined the effect of PN on the IL-6-induced cancer cell migration. As shown in Figure 5C, the effect of PN on the migration of MDA-MB-231 cells was monitored by a real-time cell analysis system for 12 h. The migration rate was quantified by RTCA software 2.0 (Roche Applied Science, Penzberg, Germany) (Figure 5D). PN abrogated IL-6-induced MDA-MB-231 cell migration while the viability of MDA-MB-231 cells was not affected during this period (Figure 5E). 

### 2.8. Parthenolide Selectively Inhibited the Growth of Cancer Cell Lines

Since STAT3 plays a key role in survival and growth of cancer cells, we examined the effects of PN on the growth of a panel of human cancer cell lines. PN induced significantly more death in MDA-MB-231, Du145, MDA-MB-468, and HCT116 cell lines (Figure 5F). We took a further look into the activation status of STAT3 in six representative cancer cells lines. As shown in Figure 5G, both the expression and phosphorylation of STAT3 were higher in the malignant cells that were sensitive to PN treatment. This data suggested that PN had the potential to selectively inhibit the growth of cancer cells through blocking the constitutively-activated STAT3.

## 3. Discussion

Although PN has been reported to inhibit JAK-STAT3 signaling pathway, its precise molecular mechanism has not been understood [23,33]. We presented evidence to demonstrate that PN was a covalent pan-JAK inhibitor. PN directly interacted with JAKs by covalently binding to specific cysteines of JAKs and inactivated their enzymatic activities.

The cysteines targeted by PN were all located in the FERM-SH2 domain of JAK2, which is necessary for the interaction between JAKs and receptors and is important for JAK activation [28,34,35,36,37,38,39]. The physical interactions between JAK2 and Gp130, however, did not seem to be affected by PN (data not shown), suggesting that PN may inactivate the JAKs mainly by changing their conformations. 

LC/MS analysis demonstrated that three cysteines, Cys178, Cys243, and Cys480, of JAK2 were covalently modified by PN. We noticed from the protein sequence alignment that Tyk2 seemed to have only one of the three PN-targeted cysteines, the Cys536, the JAK2-Cys480 equivalent. It is, therefore, possible that a Tyk2 mutant at Cys536 may lose the inhibitory effect of parthenolide. 

The binding of PN to the JAKs were quite selective, although not specific. The human JAK2 contains 27 cysteines and PN only bound to three of them. The computational analyses of the PN-JAK2 interaction predicted the binding preference for Cys243 and Cys480, which is consistent with our experimental data. The binding of PN to Cys178 is somewhat unexpected because cys178 is buried inside of the protein according to the crystal structure of JAK2. There is a possibility that a protein misfolding occurred during the overexpression and preparation of JAK2 so that certain cysteines, such as cys178, might become accessible to PN. PN also preferred JAKs over abundant proteins, such as tubulin and actin, and had little inhibitory effects on the kinase activities of PI3K, c-Src, MAPK1, EGFR, VEGFR3, and IGF-IR. Therefore, an appropriate tertiary structure of JAKs may be required for PN to interact with specific cysteines [40]. We also noticed that the phosphorylation of EGFR was slightly up-regulated by PN. It was reported that activation of EGFR was associated with ROS production, which transiently inactivates protein tyrosine phosphatases to enhance or prolong EGFR activation [41]. Thus, it is possible that the PN-induced up-regulation of EGFR phosphorylation was the result of PN-induced ROS.

Nucleophilic methylene-γ-lactone rings of natural compounds have often been reported to induce ROS in cells. PN also contains the methylene-γ-lactone ring and has the ability to induce ROS, which has been reported to regulate STATs signaling [42,43]. We, therefore, analyzed the roles of the PN-induced ROS in the inhibition of JAK/STAT3 signaling by PN. We found that the PN-induced ROS did not contribute to the PN inhibition of STAT3 phosphorylation in the MDA-MB-231 cells. This discrepancy may be due to different cellular contexts. Similar data has been reported that an oxidative stress caused by hydrogen peroxide treatment resulted in the inhibition of STAT phosphorylation in neuronal cells, but not in non-neuronal cells. The activation of Src in the non-neuronal cells could be a possible mechanism [44].

In summary, our study demonstrated PN as a novel covalent pan-JAK inhibitor. Elucidating the molecular mechanism of PN in inhibiting the JAK/STAT signaling pathway will contribute to therapeutic developments of JAK inhibitors and will help to make better use of PN in anti-inflammation and anti-cancer therapy.

## 4. Materials and Methods

### 4.1. Cell Culture

HepG2/STAT3 cells are gifts from Professor Xin-Yuan Fu (National University of Singapore, Singapore), which were stably transfected with STAT3-responsive firefly luciferase reporter plasmid. All other cell lines were obtained from the American Type Culture Collection. MEF, HEK293, Hela, Hs578t, HBE, H4, MDA-MB-453 cells were cultured in DMEM (Gibco, Grand Island, NY, USA) supplemented with 10% FBS (Gibco, Grand Island, NY, USA). MDA-MB-231, MDA-MB-468, HCT116, HT-29, Lovo, NCI-H1299, Colo205, BGC, H460 cells were grown in RPMI 1640 medium (Gibco, Grand Island, NY, USA) supplemented with 10% FBS. NCI-H1975 and Du145 were grown in RPMI 1640 medium (Gibco, Grand Island, NY, USA) supplemented with 10% FBS and 2 mmol/L glutamines. HepG2/STAT3 cells were cultured in MEMα medium (Gibco, Grand Island, NY, USA) supplemented with 10% FBS. All cell lines were cultured in 37 °C, 5% CO_2_, and a humidified atmosphere of 95% air.

### 4.2. Chemicals and Reagents

The following chemicals and reagents were used: parthenolide (Sigma-Aldrich, Saint Louis, MO, USA); AZD1480 (Selleck, Shanghai, China); DTT and MTT (Genebase, Shanghai, China); GSH (Shanghai Sibas Bioscience, Shanghai, China); IL-6 and IFNα (Peprotech, MN, USA); streptavidin agarose and DCFH-DA probe (Thermo Fisher Scientific, Waltham, CA, USA); total glutathione assay kit (Beyotime, Shanghai, China).

Plasmids encoding JAK2-FLAG were a gift from Prof. David E. Levy (New York University).

### 4.3. Antibodies

The following antibodies were from Cell Signaling Technology (Boston, MA, USA): phospho-EGFR (1:2000, #3777), EGFR (1:2000, #4267), phospho-InsR/IGF1R (1:2000, #3024), IGF1R (1:2000, #3018), phospho-Y705-STAT3 (1:3000, #9145), STAT3 (1:3000, #9139), STAT1 (1:2000, #9172), phospho-Y1007/1008-JAK2 (1:2000, #3776), JAK2 (1:2000, #3230), phospho-Y1022/1023-JAK1 (1:1000, #3331), JAK1 (1:2000, #3332), phospho-Y1054/1055-TYK2 (1:1000, #9321), TYK2 (1:2000, #9312), and Cofilin (1:2000, #5175). The antibodies for GP130 (1:1000, #sc-656) and α-Tubulin (1:3000, #SC-5286) were from Santa Cruz Biotechnology (Dallas, TX, USA). The antibodies for β-Actin were from Abmart (1:3000, #P30002M). The antibodies for phospho-Y1230/1231-VEGFR3 (1:2000, #CY1115) were from Cell Applications. Secondary HRP-conjugated antibodies were from Multi Sciences Biotech (1:5000, Hangzhou, China). Anti-Flag affinity gel was purchased from Bimake (1:50, #B23101, Shanghai, China).

### 4.4. Luciferase Assay

HepG2/STAT3 cells were seeded onto 96-well cell culture plates and grew to 90% confluence. Cells were then treated with PN for 1 h followed by stimulation with 10 ng/mL IL-6 for 4 h. Luciferase activity was determined using Promega luciferase kits according to the manufacturer’s instruction (Promega, Madison, WI, USA).

### 4.5. Western Blot Analysis

Western blotting was performed as previously described [45].

### 4.6. JAK2 In Vitro Kinase Assay

The JAK2 in vitro kinase assay was performed using JAK2 immunoprecipitants, an HTScan JAK2 Kinase Assay Kit (Cell Signaling Technology, Beverly, MA, USA) and streptavidin-coated 96-well plates (#22351, Beaverbio, Suzhou, China). HEK293 cells were transfected with plasmids encoding JAK2-FLAG for 24 h by Lipofectamine 2000 (Invitrogen, Carlsbad, CA, USA) and then lysed with 1 mL lysis buffer (50 mmol/L HEPES (pH 7.4), 150 mmol/L, 0.15% Triton X-100, NaCl, 0.5 mmol/L DTT, 2 mmol/L Na_3_VO_4_, 2 mmol/L NaF, 1 mmol/L PMSF, and protease inhibitor cocktail (Sigma, 1:1000)) on ice for 30 min. Lysates were centrifuged and the supernatants were immunoprecipitated with anti-Flag affinity gel. The immunoprecipitates were rinsed with kinase reaction buffer (60 mmol/L HEPES (pH 7.5), 5 mmol/L MnCl_2_, 5 mmol/L MgCl_2_, 25 μmol/L Na_3_VO_4_, 200 μmol/L ATP). JAK2 protein was incubated with PN for 30 min in kinase reaction buffer. The reaction was started by adding 1.5 μmol/L FLT3 substrate into the reaction buffer. The reaction was incubated at 25 °C for 30 min. In vitro kinase activity was conducted as described [46]. We chose to use HEK 293T cells because they are amenable to liposome-mediated transformations.

### 4.7. HPLC-MS Analysis of GSH-PN Adduct

A total of 0.5 mmol/L PN was incubated with 2 mmol/L of GSH in 20 mmol/L Tris-HCl (pH 7.5) for 1 h at 37 °C. The products were subjected to LC-MS system (ESI-positive mode; mobile phase: methanol/water (1:1 *v*/*v*); rate: 0.2 mL/min). The Agilent HP 1100 series HPLC system was from Agilent Technologies (Palo Alto, CA, USA). The LCQ Deca ion trap mass spectrometer was from Thermo Finnigan (San Jose, CA, USA).

### 4.8. Syntheses of Biotinylated Parthenolide

Biotinylated parthenolide (Bio-PN) was prepared by oxidation of parthenolide first with selenium dioxide and tert-butylhydroperoxide to furnish the allylic alcohol, as previously described [47].

### 4.9. Pull-Down Assay

MDA-MB-231 cells were grown on 100 mm dishes to 100% confluence. Then cells were pretreated with PN or DMSO for 1 h and incubated with Bio-PN or DMSO for 1 h. After that, cells were lysed with 1 mL lysis buffer (pH 7.4, 50 mmol/L Tris-HCl, 150 mmol/L NaCl, 1 mmol/L EDTA, 1% NP-40, 1 mmol/L PMSF, and protease inhibitor cocktail) on ice for 0.5 h. Lysates were centrifuged at 12,000× *g* at 4 °C for 10 min. The supernatant was incubated with 5% (*v*/*v*) streptavidin agarose beads for 2 h at room temperature. The precipitates were rinsed five times with lysis buffer and lysed with Laemmli buffer, followed by Western blot analysis.

### 4.10. In Vitro Kinase Assay

Human cSRC, EGFR, PI3K and MAPK1 in vitro kinase assays were performed by Eurofins’ KinaseProfiler service (http://www.eurofins.com/pharma-services/pharmadiscovery-services/services/in-vitro-pharmacology/kinases/biochemical.aspx).

### 4.11. HPLC–MS/MS Analysis of JAK2

HEK293 cells were transfected with plasmids encoding mouse JAK2-FLAG for 24 h by Lipofectamine 2000 (Invitrogen). Cells were then treated with 20 mmol/L PN for 1 h and lysed as described above. JAK2 protein was immunoprecipitated by anti-Flag affinity gel and harvested by Laemmli buffer. JAK2 was pre-separated by SDS-PAGE and was cut off from the PAGE and digested in gel [37]. The tryptic peptides were desalted and dried in a Speed-Vac. The dried peptides were processed as described previously [48]. Peak lists of the HPLC/MS/MS data were generated by Proteome Discoverer software (version 1.4, Thermo Fisher) and searched against the UniProt Human database by Mascot (v2.3, Matrix Science Ltd., London, UK). The protease was set to trypsin/P allowing for a maximum of two missed cleavage sites. The fixed modification of cysteine residues was set to carbamidomethylation. The variable modification was set to nature product derivatization, protein N-terminal acetylation, and methionine oxidation. The drug-modified peptide spectra with a Mascot ion score of more than 20 were manually inspected with stringent criteria, as described [38].

### 4.12. Docking Study

Docking study was conducted by Maestro 10.1. Crystal structure of JAK2 (4Z32) was downloaded from RCSB (Protein Date Bank). Protein Preparation Wizard Workflow (Schrödinger program suite) was used to prepare the protein and Ligand Preparation was used to prepare the compound. PN was docked into the defined binding site without constraint. Results were generated by Pymol based on the Prime-score.

### 4.13. Determination of Cellular ROS

Accumulation of intracellular ROS was detected with the probe DCFH-DA as described [49]. Briefly, after drug treatment, cells were incubated with 10 μmol/L DCFH2-DA in the cell culture incubator for 20 min. The labeled cells were washed and harvested. To quantify ROS, the fluorescence intensity was measured by flow cytometry (FACSCalibur, BD Biosciences, San Diego, CA, USA).

### 4.14. Determination of Cellular GSH Level

Cellular glutathione level was determined by a Total Glutathione Assay Kit (Beyotime, Shanghai, China) according to the instruction of the manufacturer.

### 4.15. MTT Assay

MTT assay was performed as previously described [45].

### 4.16. Fluorimetric Method to Determine the Viability of MDA-MB-231

MDA-MB-231 cells were seeded at 20,000 cells per well in serum-free 1640 medium with or without 50 ng/mL IL-6 in a 96-well plate. Cells were then treated with PN. Twenty microliters of 10% alamar blue was added into each well. Fluorescence was measured 4, 6, 8, 12 h later.

### 4.17. Migration Assay

MDA-MB-231 cells were seeded at 20,000 cells per well in serum-free 1640 medium with or without 50 ng/mL IL-6 in the upper chamber of CIM-plate16. And 150 µL 1640 medium with 10% FBS was added in the lower chamber of CIM-plate16. Cells were then treated with PN. Insert the CIM-plate 16 into the RTCA DP Analyzer (Roche Applied Science, Penzberg, Germany). The following steps were performed as described [50].

## 5. Conclusions

In conclusion, we have identified parthenolide as a covalent pan-JAK inhibitor that blocked STAT3 signaling by directly interacting and inactivating JAKs. Consequently, parthenolide inhibited the growth and migration of cancer cells that had constitutively activated STAT3. Therefore, parthenolide is a promising anti-inflammation and anticancer drug candidate.

## Figures and Tables

**Table 1:** Effects of 50 μmol/L parthenolide on the kinase activity of MAPK1, cSrc, PI3K, and EGFR.

Kinase	EGFR	PI3K	cSRC	MAPK1
Activity (%)	116	107	91	86

## Supplementary Materials

The following are available online.
