# Supplementary material for: Parthenolide Inhibits STAT3 Signaling by Covalently Targeting Janus Kinases
Source: Molecules. 2018 Jun 19;23(6):1478. doi: 10.3390/molecules23061478 (PMC6100543; doi:10.3390/molecules23061478)
Supplement: Supplementary file 1 [file molecules-23-01478-s001.pdf]

molecules

Parthenolide Inhibits STAT3 Signaling by Covalently Targeting Janus Kinases

Supplementary figures

Man Liu [1,2], Chengqian Xiao [3,4], Mingwei Sun [2,5], Minjia Tan [2,5], Lihong Hu [3] and Qiang Yu [1,2,*]

1 Shanghai Institute of Materia Medica, Chinese Academy of Sciences, Shanghai 201203, China; liuman@simm.ac.cn
2 University of Chinese Academy of Sciences, Beijing 100049, China; cmusmw@163.com (M.S.); mjtan@simm.ac.cn (M.T.)
3 Jiangsu Key Laboratory for Functional Substance of Chinese Medicine, Jiangsu Collaborative Innovation Center of Chinese Medicinal Resources Industrialization, Stake Key Laboratory Cultivation Base for TCM Quality and Efficacy, School of Pharmacy, Nanjing University of Chinese Medicine, Nanjing 210023, China; xchq890503@163.com (C.X.); lhhu@njucm.edu.cn (L.H.)
4 State Key Laboratory of Drug Research, Shanghai Institute of Materia Medica, Chinese Academy of Sciences, Shanghai 201203, China
5 The Chemical Proteomics Center, Shanghai Institute of Materia Medica, Chinese Academy of Sciences, Shanghai 201203, China
* Correspondence: qyu@sibs.ac.cn; Tel.: +86-021-5080-1790

Match to: **G5E852** Score: **28851**
Tyrosine-protein kinase OS=Mus musculus GN=Jak2 PE=1 SV=1
Found in search of 20170112-jak2-PN.mgf

Nominal mass (M_r): **130511**; Calculated pI value: **7.19**
NCBI BLAST search of G5E852 against nr
Unformatted sequence string for pasting into other applications

Variable modifications: Acetyl (Protein N-term),Oxidation (M),Carbamidomethyl (C),PN (C)
Cleavage by Trypsin/P: cuts C-term side of KR
Sequence Coverage: **85%**

Matched peptides shown in **Bold Red**

1 MGMACLTMTE MEATSTSPVH QNGDIPGSAN SVK**QIEPVLQ VYLYHSLGQA**

51 **EGEYLKFPSG EYVAEEICVA ASKACGITPV YHNMFALMSE TERIWYPPNH**

101 **VFHIDESTRH DILYR**IR**FYF PHWYCSGSSR** TYRYGVSR**GA EAPLLDDFVM**

151 **SYLFAQWRHD FVHGWIKVPV THETQEECLG MAVLDMMR**IA K**EKDQTPLAV**

201 **YNSVSYK**TFL PKCVR**AKIQD YHILTR**KRIR YRFR**RFIQQF SQCK**ATARNL

251 KLK**YLINLET LQSAFYTEQF EVK**ESAR**GPS GEEIFATIII TGNGGIQWSR**

301 GK**HKESETLT EQDVQLYCDF PDIIDVSIKQ ANQECSNESR IVTVHKQDGK**

351 **VLEIELSSLK EALSFVSLID GYYRLTADAH HYLCKEVAPP AVLENIHSNC**

401 **HGPISMDFAI SK**LK**KAGNQT GLYVLR**CSPK **DFNKYFLTFA VERENVIEYK**

451 **HCLITKNENG EYNLSGTKRN FSNLKDLLNC YQMETVRSDS IIFQFTKCCP**

501 **PKPKDKSNLL VFRTNGISDV QISPTLQRHN NVNQMVFHKI RNEDLIFNES**

551 **LGQGTFTKIF K**GVR**REVGDY GQLHKTEVLL KVLDKAHRNY SESFFEAASM**

601 **MSQLSHKHLV LNYGVCVCGE ENILVQEFVK FGSLDTYLKK** NK**NSINILWK**

651 LGVAK**QLAWA MHFLEEKSLI HGNVCAKNIL LIR**EEDR**RTG NPPFIKLSDP**

701 **GISITVLPKD ILQERIPWVP PECIENPKNL NLATDKWSFG TTLWEICSGG**

751 **DKPLSALDSQ RKLQFYEDKH QLPAPKWTEL ANLINNCMDY EPDFRPAFR**A

801 VIR**DLNSLFT PDYELLTEND MLPNMRIGAL GFSGAFEDRD PTQFEER**HLK

851 **FLQQLGKGNF GSVEMCRYDP LQDNTGEVVA VKKLQHSTEE HLRDFEREIE**

901 **ILKSLQHDNI VKYKGVCYSA GR**RNLR**LIME YLPYGSLR**DY LQKHKERIDH

951 K**KLLQYTSQI CKGMEYLGTK RYIHRDLATR NILVENENR**V K**IGDFGLTKV**

1001 **LPQDKEYYKV KEPGESPIFW YAPESLTESK** FSVASDVWSF GVVLYELFTY

1051 IEK**SKSPPVE FMR**MIGNDK**Q GQMIVFHLIE LLK**SNGR**LPR PEGCPDEIYV**

1101 **IMTECWNNNV SQRPSFR**DLS LR**VDQIRDSI AA**

Figure S1 JAK2 protein was well identified with 85% sequence coverage.

MS/MS Fragmentation of **VPVTHETQEECLGMAVLDMMR**
Found in **G5E852**, Tyrosine-protein kinase OS=Mus musculus GN=Jak2 PE=1 SV=1

Match to Query 13090: 2684.221212 from(895.747680,3+) intensity(36318.6602)
Title: File20882 Spectrum9689 scans: 11683
Data file TEST_151221212047.mgf

Click mouse within plot area to zoom in by factor of two about that point
Or, [Plot from] 200 to 2000 Da [Full range]
Label all possible matches ○ Label matches used for scoring ◉

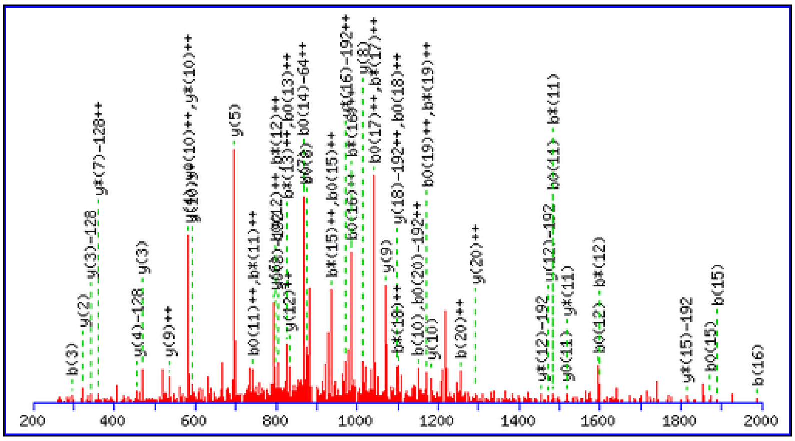

Monoisotopic mass of neutral peptide Mr(calc): 2684.2206
Variable modifications:
C11 : PN (C)
M14 : Oxidation (M), with neutral losses 0.0000(shown in table), 63.9983
M19 : Oxidation (M), with neutral losses 0.0000(shown in table), 63.9983
M20 : Oxidation (M), with neutral losses 0.0000(shown in table), 63.9983
Ions Score: 21 **Expect:** 0.38
Matches : 55/366 fragment ions using 101 most intense peaks (help)

#	b	b^{++}	b*	b^{*++}	b^0	b^{0++}	Seq.	y	y^{++}	y*	y^{*++}	y^0	y^{0++}	#
1	100.0757	50.5415					V							21
2	197.1285	99.0679					P	2586.1594	1293.5834	2569.1329	1285.0701	2568.1489	1284.5781	20
3	**296.1969**	148.6021					V	2489.1067	1245.0570	2472.0801	1236.5437	2471.0961	1236.0517	19
4	397.2445	199.1259			379.2340	190.1206	T	2390.0383	1195.5228	2373.0117	1187.0095	2372.0277	1186.5175	18
5	534.3035	267.6554			516.2929	258.6501	H	2288.9906	1144.9989	2271.9640	1136.4857	2270.9800	1135.9937	17
6	663.3461	332.1767			645.3355	323.1714	E	2151.9317	1076.4695	2134.9051	1067.9562	2133.9211	1067.4642	16
7	764.3937	382.7005			746.3832	373.6952	T	2022.8891	1011.9482	2005.8625	1003.4349	2004.8785	1002.9429	15
8	892.4523	446.7298	875.4258	438.2165	874.4417	437.7245	Q	1921.8414	961.4243	1904.8149	952.9111	1903.8308	952.4191	14
9	1021.4949	511.2511	1004.4684	502.7378	1003.4843	502.2458	E	1793.7828	897.3951	1776.7563	888.8818	1775.7723	888.3898	13
10	**1150.5375**	575.7724	1133.5109	567.2591	1132.5269	566.7671	E	1664.7402	832.8738	1647.7137	824.3605	1646.7297	823.8685	12
11	1501.6879	751.3476	1484.6614	742.8343	1483.6774	742.3423	C	1535.6976	768.3525	1518.6711	759.8392	1517.6871	759.3472	11
12	1614.7720	807.8896	1597.7454	799.3764	1596.7614	798.8844	L	***1184.5472***	592.7772	1167.5207	584.2640	1166.5367	583.7720	10
13	1671.7935	836.4004	1654.7669	827.8871	1653.7829	827.3951	G	***1071.4632***	536.2352	1054.4366	527.7219	1053.4526	527.2299	9
14	1818.8289	909.9181	1801.8023	901.4048	1800.8183	900.9128	M	***1014.4417***	507.7245	997.4151	499.2112	996.4311	498.7192	8
15	**1889.8660**	945.4366	1872.8394	936.9233	1871.8554	936.4313	A	***867.4063***	434.2068	850.3797	425.6935	849.3957	425.2015	7
16	**1988.9344**	994.9708	1971.9078	986.4576	1970.9238	985.9655	V	***796.3692***	398.6882	779.3426	390.1750	778.3586	389.6829	6
17	2102.0184	1051.5129	2084.9919	1042.9996	2084.0079	1042.5076	L	***697.3008***	349.1540	680.2742	340.6407	679.2902	340.1487	5
18	2217.0454	1109.0263	2200.0188	1100.5131	2199.0348	1100.0210	D	***584.2167***	292.6120	567.1901	284.0987	566.2061	283.6067	4
19	2364.0808	1182.5440	2347.0542	1174.0308	2346.0702	1173.5387	M	***469.1898***	235.0985	452.1632	226.5852			3
20	2511.1162	1256.0617	2494.0896	1247.5485	2493.1056	1247.0564	M	***322.1544***	161.5808	305.1278	153.0675			2
21							R	175.1190	88.0631	158.0924	79.5498			1

Figure S2 Cys178 of mouse JAK2 protein was covalently modified by parthenolide.

MS/MS Fragmentation of **QANQECSNESR**
Found in **G5E852**, Tyrosine-protein kinase OS=Mus musculus GN=Jak2 PE=1 SV=1

Match to Query 7546: 1512.650608 from(757.332580,2+) intensity(633753.4375)
Title: File20882 Spectrum6100 scans: 7377
Data file TEST_151221212047.mgf

Click mouse within plot area to zoom in by factor of two about that point
Or, Plot from 200 to 1500 Da Full range
Label all possible matches Label matches used for scoring

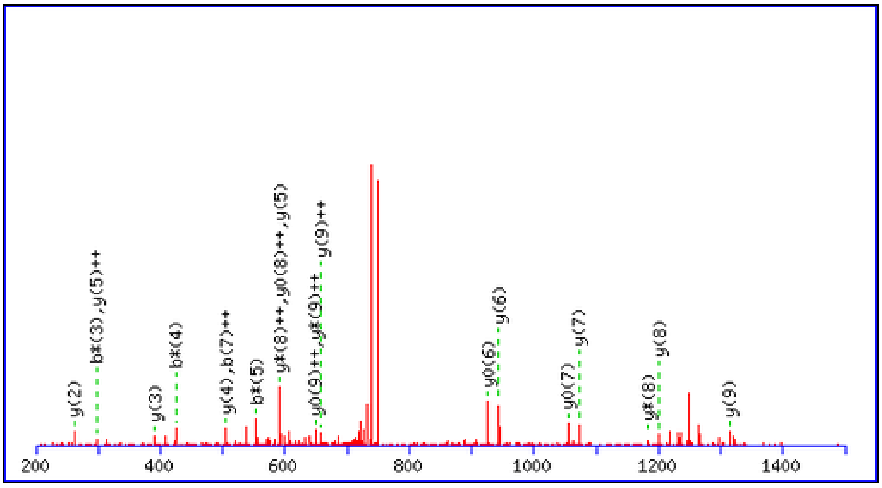

Monoisotopic mass of neutral peptide Mr(calc): 1512.6514
Variable modifications:
C6 : PN (C)
Ions Score: 52 **Expect:** 9e-005
Matches : 21/110 fragment ions using 25 most intense peaks (help)

#	b	b++	b*	b*++	b0	b0++	Seq.	y	y++	y*	y*++	y0	y0++	#
1	129.0659	65.0366	112.0393	56.5233			Q							11
2	200.1030	100.5551	183.0764	92.0418			A	1385.6002	693.3037	1368.5736	684.7904	1367.5896	684.2984	10
3	314.1459	157.5766	297.1193	149.0633			N	***1314.5631***	657.7852	1297.5365	649.2719	1296.5525	648.7799	9
4	442.2045	221.6059	425.1779	213.0926			Q	***1200.5201***	600.7637	1183.4936	592.2504	1182.5096	591.7584	8
5	571.2471	286.1272	554.2205	277.6139	553.2365	277.1219	E	***1072.4616***	536.7344	1055.4350	528.2211	1054.4510	527.7291	7
6	922.3975	461.7024	905.3709	453.1891	904.3869	452.6971	C	***943.4190***	472.2131	926.3924	463.6998	925.4084	463.2078	6
7	1009.4295	505.2184	992.4030	496.7051	991.4190	496.2131	S	***592.2685***	296.6379	575.2420	288.1246	574.2580	287.6326	5
8	1123.4725	562.2399	1106.4459	553.7266	1105.4619	553.2346	N	***505.2365***	253.1219	488.2100	244.6086	487.2259	244.1166	4
9	1252.5150	626.7612	1235.4885	618.2479	1234.5045	617.7559	E	***391.1936***	196.1004	374.1670	187.5871	373.1830	187.0951	3
10	1339.5471	670.2772	1322.5205	661.7639	1321.5365	661.2719	S	***262.1510***	131.5791	245.1244	123.0659	244.1404	122.5738	2
11							R	175.1190	88.0631	158.0924	79.5498			1

Figure S3 Cys335 of mouse JAK2 protein was covalently modified by parthenolide.

MS/MS Fragmentation of **DLLNCYQMETVR**
Found in **G5E852**, Tyrosine-protein kinase OS=Mus musculus GN=Jak2 PE=1 SV=1

Match to Query 9298: 1747.818088 from(874.916320,2+) intensity(46608.8789)
Title: File20882 Spectrum11135 scans: 13419
Data file TEST_151221212047.mgf

Click mouse within plot area to zoom in by factor of two about that point
Or, Plot from 200 to 1700 Da Full range
Label all possible matches ○ Label matches used for scoring ◉

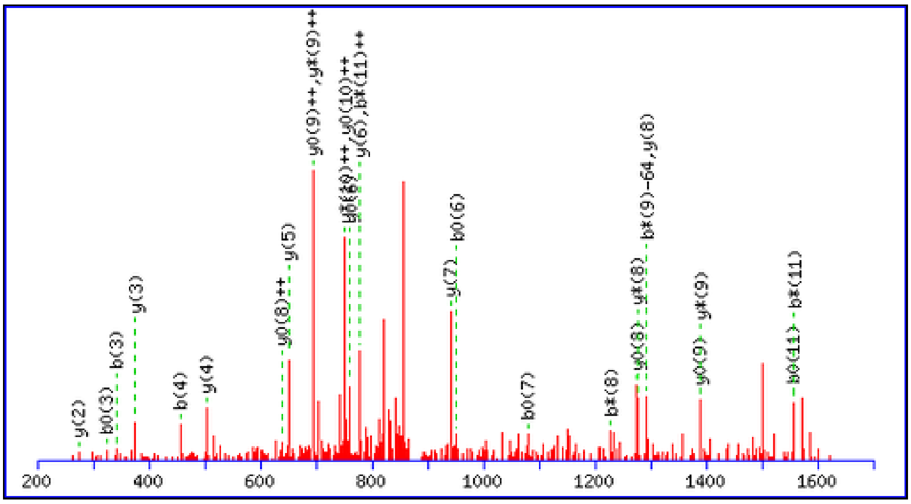

Monoisotopic mass of neutral peptide Mr(calc): 1747.8161
Variable modifications:
C5 : PN (C)
M8 : Oxidation (M), with neutral losses 0.0000(shown in table), 63.9983
Ions Score: 34 **Expect:** 0.022
Matches : 27/188 fragment ions using 42 most intense peaks (help)

#	b	b^{++}	b*	b^{*++}	b^0	b^{0++}	Seq.	y	y^{++}	y*	y^{*++}	y^0	y^{0++}	#
1	116.0342	58.5207			98.0237	49.5155	D							12
2	229.1183	115.0628			211.1077	106.0575	L	1633.7964	817.4019	1616.7699	808.8886	1615.7859	808.3966	11
3	**342.2023**	171.6048			324.1918	162.5995	L	1520.7124	760.8598	1503.6858	752.3465	1502.7018	751.8545	10
4	**456.2453**	228.6263	439.2187	220.1130	438.2347	219.6210	N	1407.6283	704.3178	1390.6018	695.8045	1389.6177	695.3125	9
5	807.3957	404.2015	790.3692	395.6882	789.3851	395.1962	C	***1293.5854***	647.2963	1276.5588	638.7831	1275.5748	638.2910	8
6	970.4590	485.7332	953.4325	477.2199	952.4485	476.7279	Y	***942.4349***	471.7211	925.4084	463.2078	924.4244	462.7158	7
7	1098.5176	549.7624	1081.4911	541.2492	1080.5070	540.7572	Q	***779.3716***	390.1894	762.3451	381.6762	761.3611	381.1842	6
8	1245.5530	623.2801	1228.5265	614.7669	1227.5424	614.2749	M	***651.3130***	326.1602	634.2865	317.6469	633.3025	317.1549	5
9	1374.5956	687.8014	1357.5691	679.2882	1356.5850	678.7962	E	***504.2776***	252.6425	487.2511	244.1292	486.2671	243.6372	4
10	1475.6433	738.3253	1458.6167	729.8120	1457.6327	729.3200	T	***375.2350***	188.1212	358.2085	179.6079	357.2245	179.1159	3
11	1574.7117	787.8595	1557.6851	779.3462	1556.7011	778.8542	V	***274.1874***	137.5973	257.1608	129.0840			2
12							R	175.1190	88.0631	158.0924	79.5498			1

Figure S4 Cys480 of mouse JAK2 protein was covalently modified by parthenolide.
